# Comparison of two software programs used to determine the relative supersaturation of urine ions

**DOI:** 10.3389/fvets.2023.1146945

**Published:** 2023-06-02

**Authors:** Reshma M. Anthony, Stephen Davidson, Jennifer M. MacLeay, John Brejda, Peter Werness, Dennis E. Jewell

**Affiliations:** ^1^Hill’s Pet Nutrition, Inc., Topeka, KS, United States; ^2^Alpha Statistical Consulting, Lincoln, NE, United States; ^3^Consultant for Hill’s Pet Nutrition, Inc, Topeka, KS, United States; ^4^Department of Grain Science and Industry, Kansas State University, Manhattan, KS, United States

**Keywords:** cats, dogs, EQUIL software, calcium oxalate, struvite, uroliths, stone risk

## Abstract

**Introduction:**

Relative supersaturation (RSS) values for urine crystals are a measure of the risk of urinary stone formation and have been shown to be lowered in foods shown to aid in the management of urolithiasis. In order to calculate RSS in pets, computer programs have been developed to calculate RSS and aid in the understanding of stone formation in veterinary medicine. However, some older programs have not been updated for use in animals, and the specific coefficients used are not publically available. One of the first RSS programs was developed in BASIC computer language and published in 1985 which was called EQUIL2. The EQUIL2 program was updated to a compiled version compatible with a PC platform. However, the formulas could not be read or altered.

**Methods:**

This study evaluates a new program with known coefficients to the original EQUIL2 program. The RSS values of the two programs were compared through a *t*-test, calculating the r^2^ from correlation analysis, Lin’s concordance correlation coefficient, and by a Bland–Altman analysis of outputs from the two programs using urine samples from healthy dogs and cats.

**Results and Discussion:**

Our results show that for both magnesium ammonium phosphate (struvite) and calcium oxalate, the RSS values of the original program could be calculated from the new programs RSS values. Although the actual RSS values were different (as might be expected through the use of the updated coefficients and different thermodynamic stability constants in the calculations) the results were highly correlated, finding elevations and reductions in RSS proportionally in the same urine samples. The current work creates a foundation for using the modernized program to calculate RSS and provides a shared method for understanding the risk of struvite and calcium oxalate stone formation.

## 1. Introduction

Uroliths are abnormal mineral (stony) deposits occurring in the urinary tract. Stones form by precipitation when precursor molecules exceed their saturation level in the urine and inhibitors fail to prevent agglomeration into substantial calculi. The saturation level is the maximum amount of solute that can be dissolved in a solvent at a specific temperature and is influenced by the solvents pH, its milieu of ions and factors influencing precipitation. Within the urinary system of a living mammal, there is an absence of large temperature fluctuations, so the saturation level is mainly dependent on the pH of the solution (urine) and the concentration of the specific molecules. A relative supersaturated solution occurs when more than the usual amount of solute required to form crystals is dissolved in the solution at a stable temperature; this phenomenon occurs when pH changes increase the solubility of the solute as well as the interaction of the molecules in the system serving to stabilize the solution. Therefore, relative supersaturation is defined as the ratio of the actual concentration of a dissolved solute in urine to its theoretical solubility in water (1). The RSS ratio is a unitless number reflecting urine pH and specific ion concentration, taking into account the thermodynamic stability constants and their interaction.

Urine becomes supersaturated when it contains more material than can be dissolved by water under normal circumstances. Supersaturation is required for stone formation; however, a relative supersaturation above 1 in companion animals’ canine and feline urine may or may not result in stone formation (2–4).

Urolithiasis is common in humans, affecting about 9% of the population in the United States (5), while nephroliths are over 5% (6) and urolithiasis is a significant disease globally (7). Urolithiasis is also prevalent in dogs and cats, causing morbidity, and in some cases, mortality. These stones commonly cause lower urinary tract disease (LUTD) in dogs and cats. Urolithiasis accounts for 18% of LUTDs in dogs (8) and about 7–22% of cases in cats (9–11). Struvite and calcium oxalate (CaOx) comprise most stones in dogs and cats (12).

Relative supersaturation (RSS) is a method for measuring the risk of specific crystal formation in urine and may help predict the risk of urolith formation. The RSS method uses mathematical determinations to predict the predisposition to form stones by analyzing urine pH and the concentrations of different minerals and compounds known to influence the formation of crystals. Risk factors for stone formation include breed, age and genetic makeup. However the controllable food choice and feeding practices also play significant roles (13–17). There are specific changes in food that can influence stone risk including fatty acids (18), hydroxyproline and starch consumption (19), salt consumption (15), and water intake (20). Also, it has been reported that feeding a specific food with the attribute of a reduced urine RSS is beneficial for the management of uroliths (14, 21, 22). Understanding the contribution that food makes in either resolving or avoiding stone formation has been an area of particular interest to pet food manufacturers for decades (1).

There are several methods proposed for calculating urine supersaturation. An ion-activity product index has been developed (23) for estimating urinary CaOx saturation. Marshall and Robertson (24) developed the nomogram for estimation of saturation of urine for different solutes such as CaOx, calcium phosphate, magnesium ammonium phosphate (MgNHPO_4_·H_2_O; struvite), uric acid, and others. The SUPERSAT program was developed in 1969 (25). An empirical activity product ratio (APR) was also developed and refined (26, 27). The EQUIL2 program (Control for this study) was developed as the first computer program for calculating urinary saturation (28) in order to provide a computerized analysis of supersaturation indices for ions in urine for routine clinical applications. The EQUIL2 program which was previously described for estimating urinary saturation (28, 29) has also been applied to evaluate the RSS in pet urine (13, 30–32).

The EQUIL2 method of computation uses urine pH and the total concentrations of 13 major urinary ions, Na, K, Ca, Mg, NH_4_, PO_4_, SO_4_, oxalate, citrate, urate, pyrophosphate, CO_2,_ and Cl, together with their thermodynamic stability constants, to calculate the free ion activity products of these urinary ions (28). The ratio of free ion activity products to the thermodynamic solubility products of their salts is then used to calculate the relative supersaturation (RSS) of the different salts. There have been previous enhancements (29) to the EQUIL software, updating the thermodynamic constants, and taking into account CaOx monohydrate’s complex chemistry. The EQUIL2 program has been utilized in various applications, including clinical evaluation of human patients suspected of stones and evaluating *in vitro* assays of lithogenic materials (33–36).

In the present study, we document the RSS values of the two common uroliths in the urine of dogs and cats, namely CaOx and magnesium ammonium phosphate (struvite) computed using the new coefficients and the previously established EQUIL2 program for RSS calculations. The goal of the study was to evaluate the relationship between the reported RSS of the two programs thus allowing a comparison of the RSS estimates from EQUIL2 which has previously been used in nutritional research. Also, to provide an understanding of the program we are now making available for general use.

## 2. Materials and methods

### 2.1. Origin of urine samples

The study included urine ion analysis from 380 healthy adult beagle dogs and 494 healthy adult shorthair cats. The health status of the dogs and cats was ascertained by daily observations from animal care technicians and annual evaluations, which included a physical examination, blood work (complete hematology and serum biochemistry panels), urinalysis, and diagnostic imaging (abdominal radiographs and ultrasound). All pets were under the care of a veterinarian (an individual for the canine colony and another for the feline colony) who was consulted on and oversaw any health related concerns. Urinary tract health was under frequent evaluation as the pets urine was frequently evaluated for crystals and signs of infection. Any dog or cat with a urinary tract health-related problem or other systemic disease was not eligible for participation. The dogs and cats were maintained separately in a temperature-controlled facility with natural daylight and opportunities for environmental enrichment and exercise. All dogs and cats were fed to maintain their optimal body weight for at least 2 weeks prior to sampling. Quantities of food offered and refused were weighed and recorded daily. Drinking water was offered *ad libitum*. During the adaptation phase the dogs and cats were housed collectively by species. The dogs and cats were also housed individually in lodges, to which they had been acclimated via a habituation program, for the 24 h of urine collection. During the collection period all naturally voided urine was collected in bottles containing thymol. A minimum of 30 mL of urine was saved for analysis. Urine was held at body temperature during collection and pH was measured. Aliquots were frozen at-80°C for all other analysis. The protocols were approved by appropriate Animal Care and Use Committees and adhered to the sponsor’s Animal Welfare Policy.

### 2.2. Urine ion analysis

Urine was analyzed for Ca^+2^, Cl^−^, citrate, Mg^+2^, oxalate, PO_4_^−3^, K^+^, Na^+^, SO_4_^−2^, NH_4_^+^, and pH. The specific analyte methods were validated in the laboratory doing the analysis following the FDA Guidelines for Biochemical Analyses. The mineral content of the urine, specifically, Ca, Mg, K, Na and P, are measured using ICP-OES (AOAC Official Method 968.08, Minerals in Animal Feeds and Pet Food, Atomic Absorption Spectrophotometric Method). The minerals (Ca, Mg, K, Na, and P) are measured using an Agilent 5,100 Inductively Coupled Plasma—Optical Emission Spectrometer. The urine is prepared by acidifying 500 μL of the urine with dilute hydrochloric acid. The instrument is calibrated using traceable solutions containing each of these elements. The concentrations of chloride, sulfate and citrate are measured simultaneously using hydroxide ion exchange chromatography. Each sample of whole urine is diluted 400:1 with deionized Water (AOAC Method 993.3, Inorganic Anions in Water by Ion Chromatographic Method). The instrument is calibrated using traceable standards of each ion of interest. The concentration of oxalate in each urine sample is measured using ion exclusion chromatography. The raw urine is acidified using dilute phosphoric Acid prior to freezing to ensure that the oxalate stays in solution. The method used is similar to the previously published method (37). The instrument is calibrated using gravimetrically prepared solutions of sodium oxalate. The urine pH was measured by glass electrode (VWR SympHony B30PCI). The meter was calibrated using phosphate buffer standards at pH 4, 7, and 10. Sulfate, Chloride, and Citrate concentrations are measured using a Thermo-Dionex Ion Chromatograph using conductivity for detection and Chromeleon 7 Chromatographic software. The ammonium content is measured on whole undiluted urine using a method based on the previously published method (38). Basically, alkaline hypochlorite and phenol react with ammonia using sodium nitroprusside as a catalyst to form indophenol. The absorbance of indophenol at 595 nm is directly proportional to the ammonia concentration. The method is standardized with known solutions of ammonium chloride. Ammonium is measured using a Varian Varioskan microtiter plate reader using their SkanIt software. Oxalate is measured using an Agilent 1200 HPLC with a Diode Array Detector watching absorbance at 210 nm for response and OpenLab Chemstation version C.01.07 chromatographic software.

### 2.3. Pet health

Dogs and cats were assigned to the study if they were above 1 year of age and known to be healthy. Health was established initially by veterinary evaluation and monitored daily through observation by animal care technicians. Urinary tract health was monitored on average more than once a month through evaluation of urine for microbes, crystals or any abnormality (such as an unusual change in pH, urine specific gravity or microscopic abnormalities).

### 2.4. Statistical analysis

The relationship between values of RSS for CaOx and struvite calculated with the control and new software programs using the measured ion concentrations in urine from dogs and cats was examined using four assessments: a paired *t*-test; Lin’s concordance correlation coefficient (CCC) (39); calculation of the r^2^ statistic; and scatterplots with Bland–Altman plots (40). The residuals from the paired *t*-test were evaluated by inspection to assure they were normally distributed. Regarding the relationship between the two RSS estimates the scatterplots and the Bland–Altman plots are a visual depiction of the relationship between the different measurements. The Lin’s CCC is an assessment of agreement corrected for bias. These and other statistical tests to examine the repeatability of a measurement or whether two methods of measurement produced similar results have been discussed in detail (41). The graphical method, called a Bland–Altman plot, is a plot of the difference versus the average of the two different measures. On the y-axis reference lines are drawn at ±2 standard deviations (SD) and ± 3 SD of the difference. A Bland–Altman plot allows for assessment of the magnitude of disagreement, for error and bias. Linear regression was used to determine if the new program values can be used to predict old program values. A 95% confidence interval (CI) was calculated for the slope estimate. If the 95% CI contained 1, this was further confirmation of agreement between the two programs. However, even if the slope was significantly different from 1, the new program values may still be useful for predicting old program values. The strength of the prediction equations was ascertained using the r^2^ statistic. This statistic is a measure of the proportion of variation in old program values explained by the new program values. All statistical analyses were performed using SAS^®^, version 9.4 program (SAS Institute, Cary, NC, United States).

## 3. Results

### 3.1. Relative supersaturation in dogs

#### 3.1.1. CaOx

Table 1 gives the summary statistics of RSS values for CaOx in dog urine samples as determined by the EQUIL2 (Control) and the EQUIL-HL21 software with the new coefficients (New). Mean RSS CaOx values were significantly lower (difference = 2.29, SE = 0.12, *p* = <0.01) using the new coefficients.

**Table 1:** Calculated canine RSS for calcium oxalate using the control and new programs.

	Canine RSS calcium oxalate	
Statistic	Control	New
N^1^	378	378
Mean	15.44	13.15
SD	11.73	9.85
CV	76.0	74.9
Minimum	1.03	0.95
Median	12.675	10.764
Maximum	74.69	68.40
Difference (SE)	2.29 (0.12)	
Prob > |t|	<0.0001	

^1^Two values were removed because they exceeded 3 standard deviations from the population mean.

**Figure 1:**
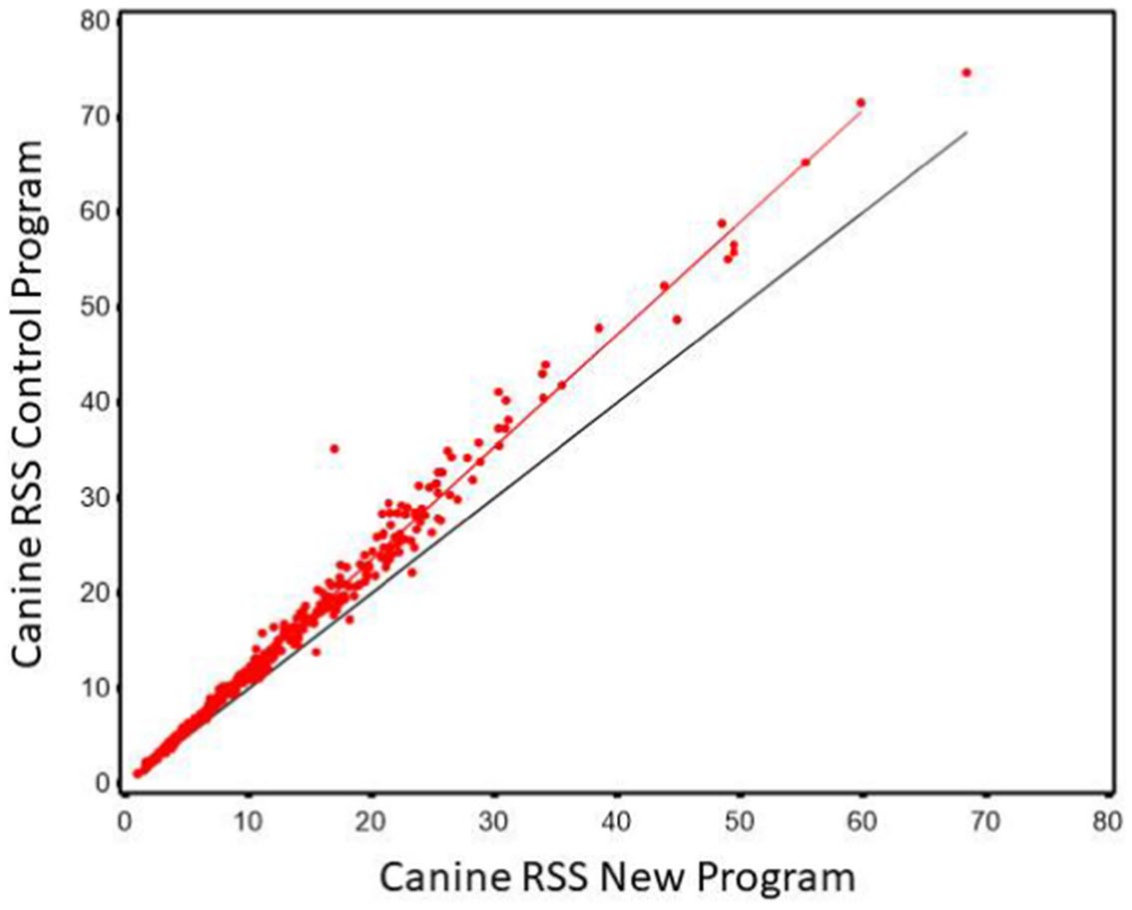
The relationship between control and new RSS calculations for calcium oxalate in canine urine. The red line indicates the best fit line from the scatter plots. The black line represents a 1:1 relationship.

Regression of CaOx values calculated using the new software program could be used to predict CaOx RSS values calculated using the control program with high precision (r^2^ of 0.985). The Lin’s CCC was 0.956 showing the agreement between the two values is high. However, the slope was significantly different from 1 (slope = 1.181, 95% CI 1.166, 1.196). This results in the average difference between the two values as shown in Figure 1. This indicates that it is at the higher RSS estimates for CaOx where the two software programs have the largest discrepancy and that they are not interchangeable.

**Figure 2:**
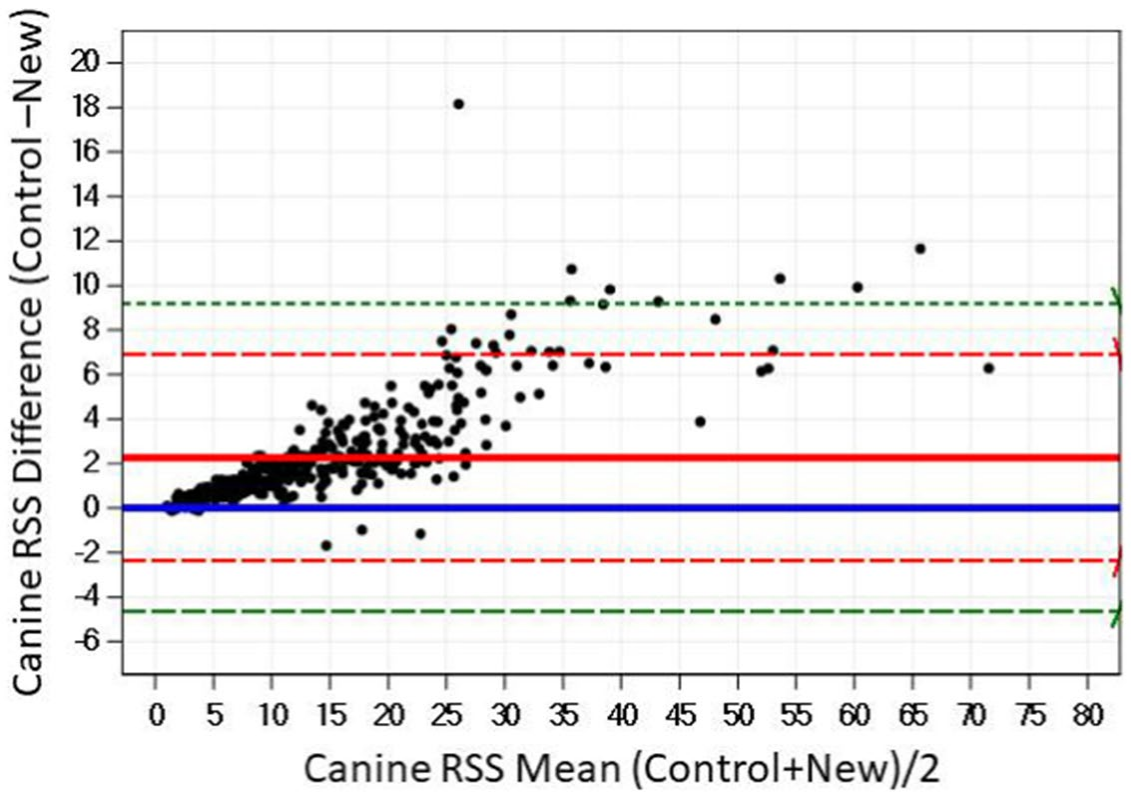
The Bland–Altman relationship between control and new RSS calculations for calcium oxalate in canine urine. The solid line indicates the mean of the difference between the values; the dashed red lines indicate 2 standard deviation limits, and the green indicate the limits at ±3 standard deviations, providing an estimate for 99% of the differences.

The Bland–Altman plots confirm the results from the regression analysis in that as RSS values for calcium oxalate increased, the difference in calculated values between the two software programs also increased (Figure 2).

#### 3.1.2. Struvite

Summary statistics of RSS values for struvite in dog urine are given in Table 2. Mean RSS values for struvite were significantly lower (difference = 4.05, SE = 0.4, *p* < 0.001) using the new software program.

**Table 2:** Calculated canine struvite RSS using the control and new programs.

	Canine RSS struvite	
Statistic	Control	New
N^1^	375	375
Mean	6.06	2.27
SD	11.30	5.95
CV	187	263
Minimum	0	0
Median	1.010	0.337
Maximum	86.56	92.78
Difference (SE)	4.05 (0.40)	
Prob > |t|	<0.0001	

**Figure 3:**
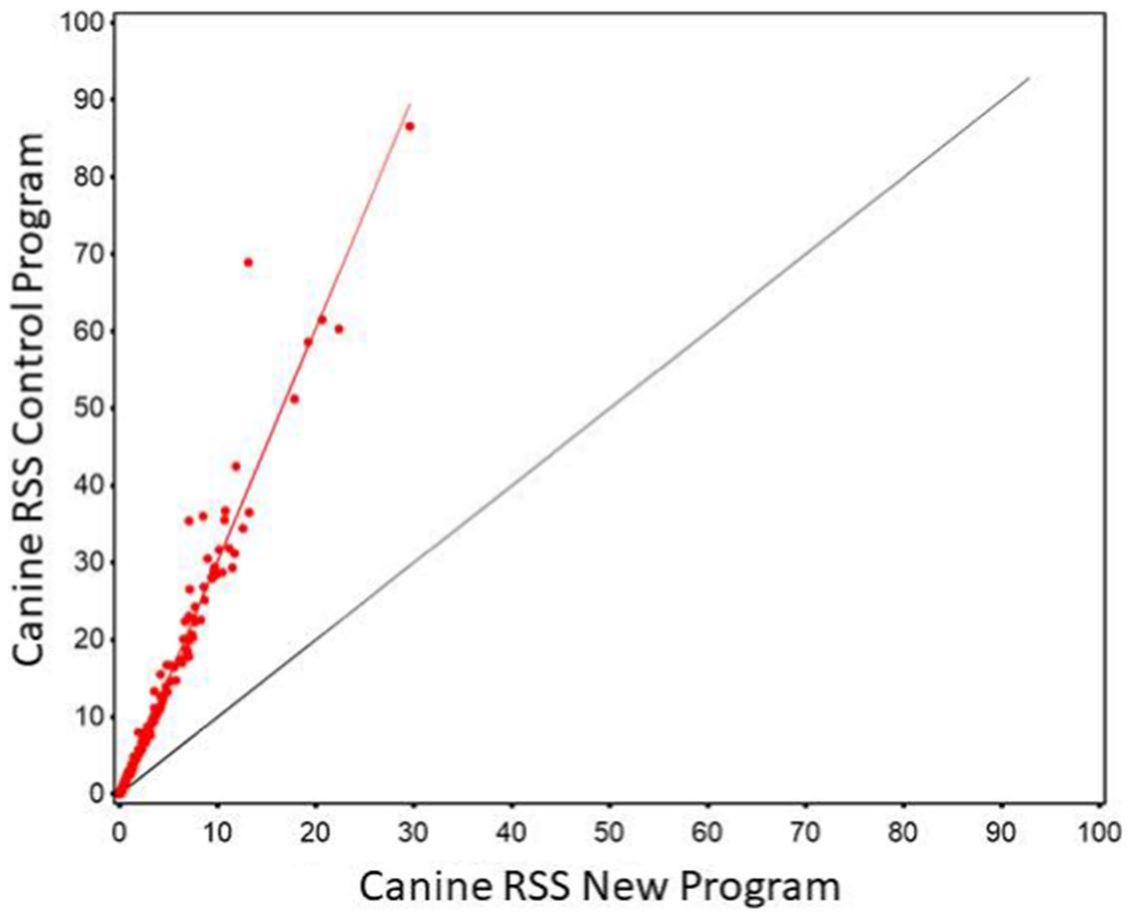
The relationship between control and new RSS calculations for struvite in canine urine. The red line indicates the best fit line from the scatter plots. The black line represents a 1:1 relationship.

Regression of struvite values calculated using the new software program could be used to predict struvite values calculated using the old software program with high precision. The strength of the prediction equation was high (*r*^2^ = 0.967). However, the slope significantly different from 1 (slope = 3.028, 95% CI 2.97, 3.09) as shown in Figure 3. The Lin’s CCC was 0.461 showing a modest agreement.

**Figure 4:**
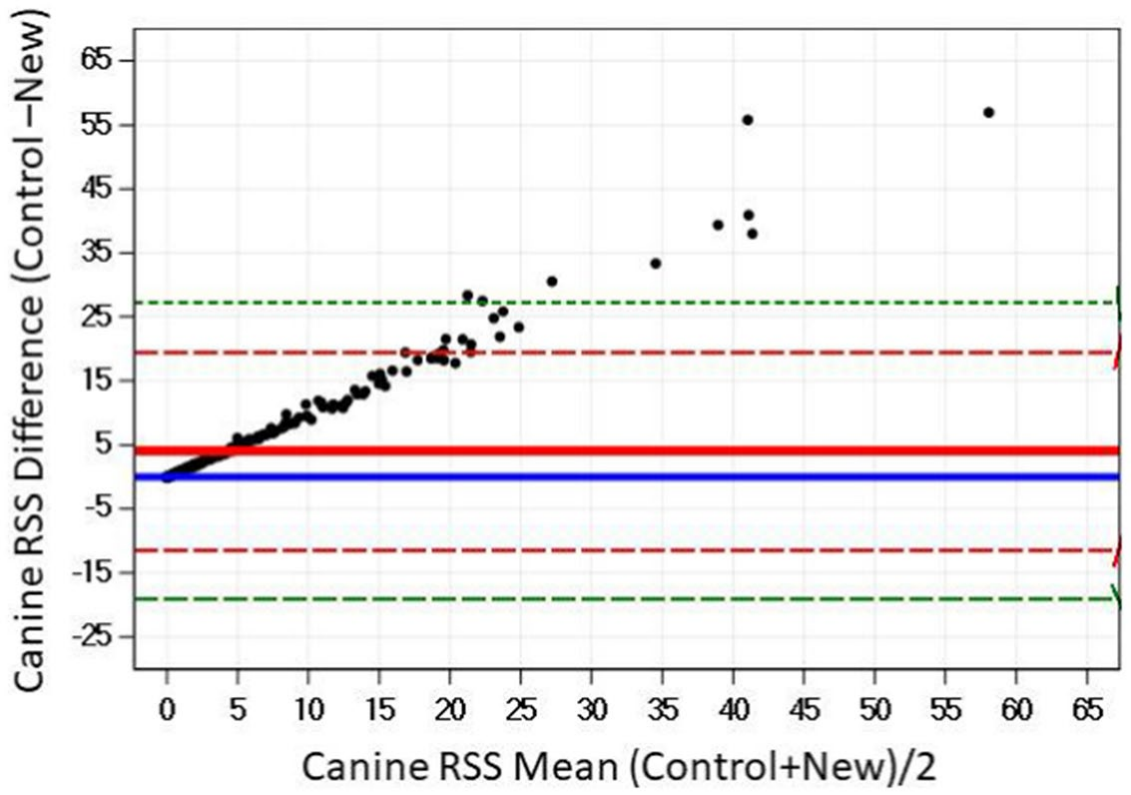
The relationship between control and new RSS calculations for struvite in canine urine. The solid red line indicates the mean of the difference between the values; the dashed red lines indicate 2 standard deviation limits, and the green indicate the limits at ±3 standard deviations, providing an estimate for 99% of the differences.

The Bland–Altman plots confirm the results from the regression analysis in that as RSS values for struvite increased, the difference in output between the two software programs also increased (Figure 4).

### 3.2. RSS in cats

#### 3.2.1. CaOx

Summary statistics of RSS values for CaOx values in cat urine samples as determined by the two software programs are given in Table 3. There was a significant difference in mean RSS values for CaOx (difference = 0.199, SE = 0.014, *p* < 0.0001) between the two software programs.

**Table 3:** Calculated feline calcium oxalate RSS for calcium oxalate using the control and new programs.

	Feline RSS calcium oxalate	
Statistic	Control	New
N^1^	488	486
Mean	3.42	3.22
SD	2.47	2.35
CV	72.4	73.0
Minimum	0.48	0.53
Median	2.775	2.67
Maximum	31.96	30.29
Difference (SE)	0.199 (0.014)	
Prob > |t|	<0.0001	

^1^Six values were removed because they exceeded 3 standard deviations from the population mean.

**Figure 5:**
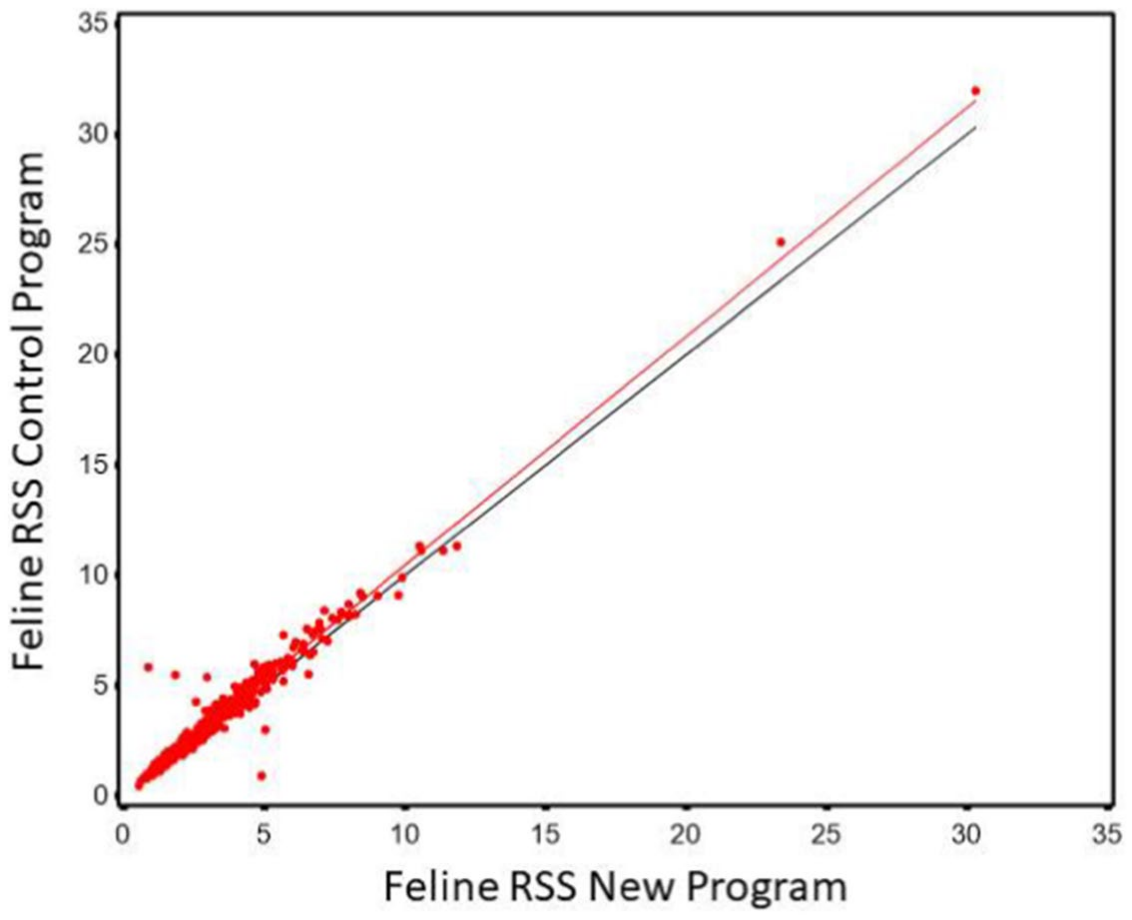
The relationship between control and new RSS calculations for calcium oxalate in feline urine. The red line indicates the best fit line from the scatter plots. The black line represents a 1:1 relationship.

As in the case with the canine the relationship of the two software programs was such that the old RSS could be predicted from the results of the new (r^2^ of 0.987) as shown in Figure 5. However, the slope was significantly different from 1 (slope = 1.048, 95% CI 1.037, 1.059). Thus, it is at the higher RSS estimates for CaOx where the two software programs have the most significant discrepancy. As in the canine CaOx RSS the Lin’s CCC showed there was a high agreement between the two values (CCC = 0.996).

**Figure 6:**
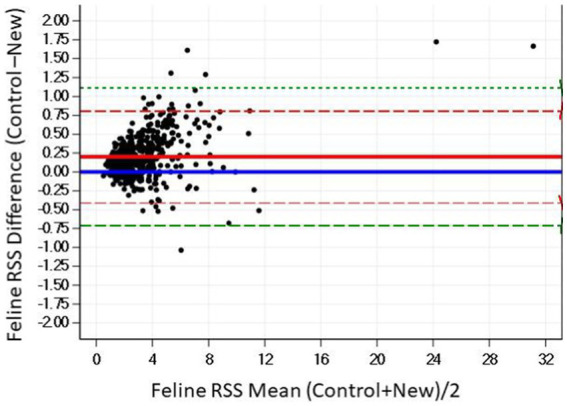
The Bland–Altman relationship between control and New RSS calculations for calcium oxalate in feline urine. The solid line indicates the mean of the difference between the values; the dashed red lines indicate 2 standard deviation limits, and the green indicate the limits at ±3 standard deviations, providing an estimate for 99% of the differences.

The Bland–Altman plots confirm the results from the regression analysis in that as RSS values were closely aligned (Figure 6).

#### 3.2.2. Struvite

Summary statistics of RSS values for struvite in cat urine are given in Table 4. Mean RSS values for struvite were significantly greater (difference = 7.1, SE = 0.61, *p* < 0.0001) from the New software as compared to the Control.

**Table 4:** Calculated feline struvite RSS using the control and new programs.

	Feline RSS struvite	
Statistic	Control	New
N	494	492
Mean	10.34	3.14
SD	19.09	5.66
CV	184.66	180.26
Minimum	0.01	0.003
Median	5.79	1.90
Maximum	296.39	88.51
Difference (SE)	7.11 (0.61)	
Prob > |t|	<0.0001	

**Figure 7:**
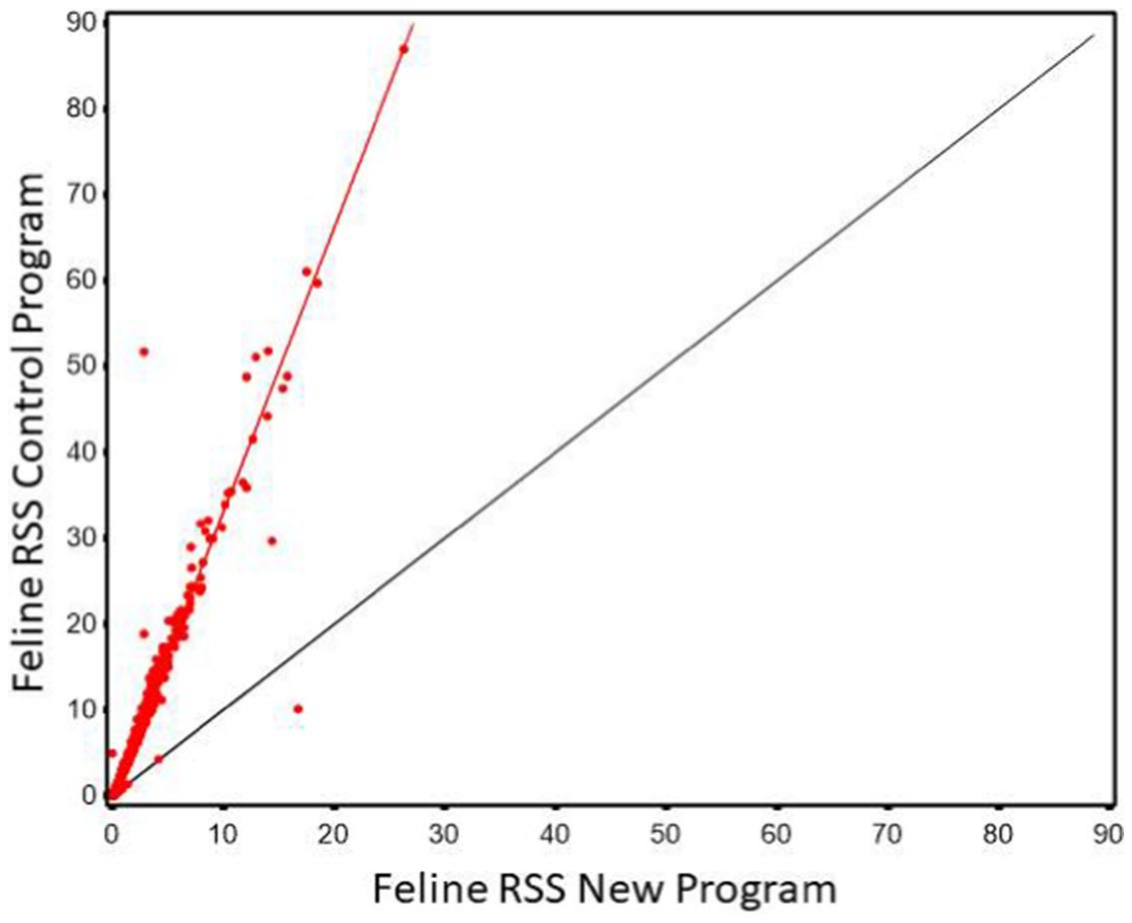
The relationship between control and new RSS calculations for struvite in feline urine. The red line indicates the best fit line from the scatter plots. The black line represents a 1:1 relationship.

Regression of MAP values calculated using the new software program could be used to predict MAP values calculated using the old software program with high precision (r^2^ of 0.971, Figure 7). However, as in the in the case with canine the slope was significantly greater than 1 (3.314, 95% CI 3.26, 3.37). The Lin’s CCC showed only modest agreement (CCC = 0.476).

**Figure 8:**
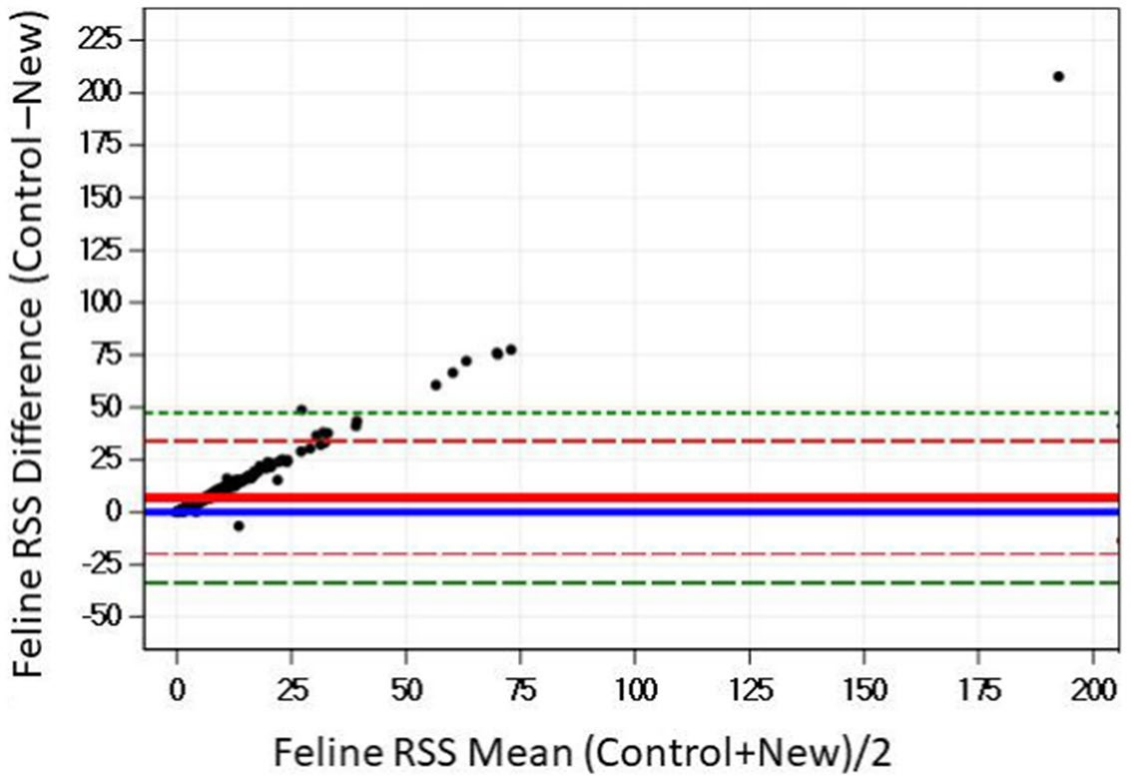
The Bland–Altman relationship between control and new RSS calculations for struvite in feline urine. The solid red line indicates the mean of the difference between the values; the dashed red lines indicate 2 standard deviation limits, and the green indicate the limits at ±3 standard deviations, providing an estimate for 99% of the differences.

The Bland–Altman plots confirms the results from the regression analysis that as RSS values for struvite increased, the difference in the output between the two software programs also increased (Figure 8).

## 4. Discussion

This study compared the two computer programs - the previously published EQUIL2 (Control) and the EQUIL-HL21 program with new coefficients (New) for calculating the relative supersaturation (RSS) of dog and cat urine with respect to CaOx and struvite. The original programs take up to 15 and 23 user-inputted ion concentrations, respectively. However, these programs are robustly designed to calculate RSS in other sample types (i.e., blood) and for multiple stone types. As such not all input variables have meaning in the urine samples or stone types considered here. This study used the analytes which are present in urine and expected to have an influence on struvite or CaOx supersaturation. The data from this work show that the two programs are highly related in that knowing the RSS from the New calculation allows one to calculate the RSS from the Control program. However, they do not produce identical results. The difference is due to the different thermodynamic stability constants used in the computations by the two programs.

The RSS values for CaOx in dogs were significantly higher using the Control program compared with the New software. This relationship generated the slope of 1.181 so that the increase was found consistently with the higher values showing the greatest differences. For the RSS of CaOx in cat urine, the agreement between the programs was good with a slope of 1.048 (Control/New). For RSS of struvite in dog urine, the slope was 3.08 with higher values in the Control program as compared to the New. Similarly, for RSS of struvite in cat urine the slope was 3.314 showing clear differences and higher values of RSS using the Control program.

We hypothesize the discrepancies in the data obtained using the two programs can be explained by the slightly different coefficients and thermodynamic stability constants used for computations between the two programs. The greater divergence in RSS at higher values indicates that a constant factor in the algorithms is responsible, although the contribution of super-anion binding sites may play a role for CaOx (42). Also, it has been shown that the thermodynamic solubility product of magnesium ammonium phosphate hexahydrate, which is used by the programs to calculate RSS for struvite, is higher than previously reported (43). This may also explain the difference in the values of RSS for struvite for the same sample computed by the two programs.

The study was designed to document the RSS values generated by the two software programs and determine the level of alignment between the values generated by both. The study therefore, does not give information on what program generates values that best represent clinical manifestations or laboratory investigations. Moreover, a limitation in this work is that urine from healthy dogs and cats, which were not known stone formers, was used to determine the range of RSS. This limits the evaluation of the algorithms. The study does provide evidence that when comparing RSS values the comparisons should be made using values calculated by the same program or through understanding the relationship between the two programs used. We also freely offer the program to researchers (Supplementary Table 1), so that well-designed, randomized, controlled clinical studies and cohort or case–control studies can be performed as desired to determine the relative effectiveness of interventions on urinary stone risk.

Comparisons between the Control program and other methods regarding their ability to predict urine supersaturation are available in the scientific literature (24, 26, 29, 44, 45). The predictive values generated by the Control program (using 10 input variables) and by the proprietary SUPERSAT program (Arup Laboratories, aruplab.com, 500 Chipeta Way, Salt Lake City, UT) or a program offered by Royal Canin, using human, dog and cat urine have been reported (39). Values for both programs were similar for CaOx but differed for struvite with the Control program calculating significantly higher struvite RSS (30). The SUPERSAT program used the 10 input variables of EQUIL2 plus uric acid. It is unlikely that the inclusion of uric acid accounts for a difference in the RSS for struvite or CaOx as uric acid only has influence of supersaturation for RSS of urate stones. Unfortunately, the SUPERSAT program and its coefficients have not been made publically available to laboratories outside the corporation that commissioned the work. LITHORISK, a new predictive software, was developed by Marangella’s group (44, 46) for calculations and graphic visualization of risk profiles for stone formation using urine samples. Data obtained from LITHORISK showed good correspondence with those obtained with the Control program in one study (46). However, another study comparing Control and LITHORISK values showed significant differences between the two programs (47). These studies collectively show that these programs can be used to predict urine supersaturation, and no one method has been established as being superior to the others. However, when comparisons are made between diagnoses, treatments, outcomes, or foods, it is essential to use RSS values generated by the same program.

This study has a limitation in that it did not use all possible analytes for predicting RSS and did not have any pets with urolithiasis as data points to evaluate what RSS prediction value is associated with stone formation. Furthermore, there is the general limitation of all RSS values as actual stone formation is dependent on nucleation and the surface energy of the solid phases of stone-salts (48, 49).

In conclusion, this study shows that the relative supersaturation of dog and cat urine with respect to CaOx or struvite can be calculated using either of these programs. Although the programs are highly related they are not interchangeable. The advantage of the new program is that it is compatible with modern operating systems and can serve as an open-source platform for use by the veterinary research community.

## Data availability statement

The raw data supporting the conclusions of this article will be made available by the authors, without undue reservation.

## Ethics statement

The animal study was reviewed and approved by the Institutional Animal Care and Use Committee of Hill’s Pet Nutrition, Inc. Written informed consent was obtained from the owner for the participation of the animals in this study.

## Author contributions

JB was responsible for statistical analysis. PW was responsible for the coefficients used and the new program to calculate relative super saturation. All authors contributed to the writing and editing of the manuscript and agreed to be accountable for the content of the work.

## Funding

This research was funded by Hill’s Pet Nutrition, Inc. The funding agency provided funds for the consultants and the bio-archive data for analysis.

## Conflict of interest

RA, SD, JM, PW, and DJ were employed by Hill’s Pet Nutrition, Inc. JB was employed by Alpha Statistical Consulting.

## Publisher’s note

All claims expressed in this article are solely those of the authors and do not necessarily represent those of their affiliated organizations, or those of the publisher, the editors and the reviewers. Any product that may be evaluated in this article, or claim that may be made by its manufacturer, is not guaranteed or endorsed by the publisher.
