# Supplementary material for: Comparison of two software programs used to determine the relative supersaturation of urine ions
Source: Front Vet Sci. 2023 Jun 2;10:1146945. doi: 10.3389/fvets.2023.1146945 (PMC10274322; doi:10.3389/fvets.2023.1146945)
Supplement: Supplementary file 1 [file Table_1.DOCX]

Supplementary Table 1. The New program.

Sub maineq()

1 Rem    ****   EQUIL   ****

2 Rem    *******************

3 Rem    MODIFIED BASIC VERSION by P. WERNESS

4 Rem    ORIGINAL *EQUIL* by B. FINLAYSON

5 Rem 22-Jan-17 version for HPN.  Reads and writes to front spreadsheet.  Checks for blank cell for C02, Urate and pyrophospate.  If blank set to 0.000001

6 Rem  7-Sep-17 version for HPN Stephen spreadsheet

7 Rem

8 Rem

9 Rem

10 Dim S(65)

11 Rem

12 Rem

14 Rem

15 Rem

16 Rem

17 Rem

18 Rem

20 S(1) = 1730000000000#

30 S(2) = 14900000#

40 S(3) = 162

50 S(4) = 145.5

60 S(5) = 20750

70 S(6) = 2640000#

80 S(7) = 55210

90 S(8) = 1247

100 S(9) = 278000

110 S(10) = 12.9

120 S(11) = 5.433

130 S(12) = 13.4

140 S(13) = 8.5

150 S(14) = 216

160 S(15) = 33.1

170 S(16) = 251.2

180 S(17) = 10

190 S(18) = 8.831

200 S(19) = 13.4

210 S(20) = 12.6

220 S(21) = 143

230 S(22) = 3597000#

240 S(23) = 685

250 S(24) = 31.3

260 S(25) = 229.6

270 S(26) = 2746

280 S(27) = 17.3

290 S(28) = 71.4

300 S(29) = 60000

310 S(30) = 505.2

320 S(31) = 12.5

330 S(32) = 3460000#

340 S(33) = 1014

350 S(34) = 31.9

360 S(35) = 188.4

370 S(36) = 4020

380 S(37) = 4.75

390 S(38) = 5.93

400 S(39) = 69900

410 S(40) = 316.7

420 S(41) = 5

430 S(42) = 10

440 S(43) = 12.9

450 S(44) = 13

460 S(45) = 8.5

470 S(46) = 58800000#

480 S(47) = 2440000000#

490 S(48) = 4970000#

500 S(49) = 171

510 S(50) = 7.05

520 S(51) = 562000

530 S(52) = 5500

540 S(53) = 794000000#

550 S(54) = 23.1

560 S(55) = 380.19

570 S(56) = 19770000#

580 S(57) = 1995000000#

590 S(58) = 55.6

600 S(59) = 1940000#

610 S(60) = 16600000000#

620 S(61) = 0.00123

630 S(62) = 18.6

640 S(63) = 1.03

650 S(64) = 1897

660 S(65) = 946

670 Dim A(120)

680 Dim T(11)

692 GoTo 742

695 Rem rem print "TITLE"; : INPUT P$

700 Rem rem print "NA"; : INPUT A(1)

702 Rem rem print "K"; : INPUT A(2)

704 Rem rem print "CA"; : INPUT A(3)

706 Rem rem print "MG"; : INPUT A(4)

708 Rem rem print "PYRO"; : INPUT A(11)

710 Rem rem print "NH4"; : INPUT A(5)

712 Rem rem print "CL"; : INPUT A(91)

714 Rem rem print "CO2"; : INPUT A(31)

716 Rem rem print "P"; : INPUT A(6)

718 Rem rem print "S"; : INPUT A(7)

720 Rem rem print "CIT"; : INPUT A(9)

722 Rem rem print "OX"; : INPUT A(8)

724 Rem rem print "PH"; : INPUT A(24)

726 Rem rem print "UR"; : INPUT A(10)

730 Rem goto 820

740 Rem pick row

742 Rem Cells(15, 14) = 123123: Stop

744 i = 29

745 j = 29

746 Rem: GoTo 780

748 Rem j = j + 1

760 A(24) = Cells(i, 2): Rem Cells(i, 14) = Cells(i, 2): Rem pH

762 A(5) = Cells(i, 3): Rem Cells(i, 15) = Cells(i, 3): Rem NH4

764 A(1) = Cells(i, 4): Rem Cells(i, 16) = Cells(i, 4): Rem Na

766 A(2) = Cells(i, 5): Rem Cells(i, 17) = Cells(i, 5): Rem K

768 Rem A(91)= 0.00270

770 A(3) = Cells(i, 7): Rem Cells(i, 19) = Cells(i, 7): Rem Ca

772 A(6) = Cells(i, 8): Rem Cells(i, 20) = Cells(i, 8): Rem P

774 A(4) = Cells(i, 9): Rem Cells(i, 21) = Cells(i, 9): Rem Mg

776 A(8) = Cells(i, 10): Rem Cells(i, 22) = Cells(i, 10): Rem Ox

778 A(9) = Cells(i, 11): Rem Cells(i, 23) = Cells(i, 11): Rem Cit

779 If A(9) = "" Then A(9) = 0.000001 Else GoTo 780

780 A(7) = Cells(i, 12): Rem Cells(i, 24) = Cells(i, 12): Rem S

781 A(91) = Cells(i, 6): Rem Cells(i, 18) = Cells(i, 6): Rem Cl

782 A(10) = "": Rem A(10) = Cells(2, 18): Cells(i, 15) = Cells(i, 18): Rem Urate

783 If A(10) = "" Then A(10) = 0.000001

784 A(11) = "": Rem A(11) = Cells(i, 29): Cells(i, 15) = Cells(i, 29): Rem Pyro

785 If A(11) = "" Then A(11) = 0.000001

786 A(31) = "": Rem A(31) = Cells(i, 20): Cells(i, 15) = Cells(i, 20): Rem C02

787 If A(31) = "" Then A(31) = 0.000001

788 Rem A(91) = Cells(i, 6): Cells(i, 18) = Cells(i, 6): Rem Cl

820 A(26) = 10 ^ (0# - A(24))

822 Rem If A(9) = "" Then A(9) = 0.000001

830 Rem Next i

840 Rem f = 2 ^ 4: Cells(11, 16) = f

850 F1 = 0.7

852 Rem Cells(11, 17) = "line 852"

860 F2 = 0.3

862 Rem Cells(11, 18) = "line 862"

870 F3 = 0.1

880 F4 = 0.02

882 Rem Cells(11, 18) = "line 882"

890 O0 = 0

900 O1 = 0

910 O2 = 0

920 O3 = 0

930 O4 = 0

940 For i = 1 To 10

942 Rem Cells(11, 19) = "line 942"

950 A(12 + i) = 0.1 * A(i)

952 Rem Cells(11, 21) = "line 952"

953 Rem Cells(11, 22) = "line 953"

960 Next i

962 Rem Cells(11, 23) = "line 962"

970 A(12) = 0.1 * A(11)

980 A(23) = 0.01 * A(12)

982 Rem Cells(11, 24) = "line 982"

990 For i = 1 To 50

992 Rem Cells(11, 25) = "line 992"

1000 A(25) = 10 ^ (0 - 13.593 + A(24))

1010 A(27) = S(1) * A(26) * A(18) * F3 / F2

1020 A(28) = S(2) * A(26) * A(27) * F2 / F1

1030 A(29) = S(3) * A(26) * A(28) * F1

1040 A(30) = S(61) * A(31) * S(58)

1050 A(32) = A(30) / (A(26) * S(59) * F1 * F1)

1060 A(33) = A(32) / (A(26) * S(60) * F2)

1070 A(34) = S(62) * A(13) * A(33) * F2

1080 A(35) = S(63) * A(13) * A(34) * F1 * F1

1090 A(36) = S(64) * A(15) * A(33) * F2 * F2

1100 A(37) = S(65) * A(16) * A(33) * F2 * F2

1110 A(38) = S(4) * A(26) * A(19) * F2 / F1

1120 A(39) = S(5) * A(26) * A(20) * F2 / F1

1130 A(40) = S(6) * A(26) * A(21) * F3 / F2

1140 A(41) = S(7) * A(26) * A(40) * F2 / F1

1150 A(42) = S(8) * A(26) * A(41) * F1

1160 A(43) = S(9) * A(26) * A(22) * F1

1170 A(44) = S(10) * A(13) * A(27) * F2

1180 A(45) = S(11) * A(13) * A(19) * F2

1190 A(46) = S(12) * A(13) * A(20) * F2

1200 A(47) = S(13) * A(13) * A(21) * F3 * F1 / F2

1210 A(48) = S(14) * A(13) * A(12) * F1 * F4 / F3

1220 A(49) = S(16) * A(13) * A(48) * F1 * F3 / F2

1230 A(50) = S(15) * A(13) * A(23) * F1 * F3 / F2

1240 A(51) = S(17) * A(14) * A(27) * F2

1250 A(52) = S(18) * A(14) * A(19) * F2

1260 A(53) = S(19) * A(14) * A(20) * F2

1270 A(54) = S(20) * A(14) * A(21) * F3 * F1 / F2

1280 A(55) = S(21) * A(14) * A(12) * F1 * F4 / F3

1290 A(56) = S(22) * A(15) * A(18) * F3 * F2 / F1

1300 A(57) = S(23) * A(15) * A(27) * F2 * F2

1310 A(58) = S(24) * A(15) * A(28) * F2

1320 A(59) = S(25) * A(15) * A(19) * F2 * F2

1330 A(60) = S(26) * A(15) * A(20) * F2 * F2

1340 A(61) = S(28) * A(15) * A(60)

1350 A(62) = S(27) * A(60) * A(20)

1360 A(63) = S(29) * A(15) * A(21) * F3 * F2 / F1

1370 A(64) = S(30) * A(15) * A(40) * F2 * F2

1380 A(65) = S(31) * A(15) * A(41) * F2

1390 A(66) = S(32) * A(16) * A(18) * F3 * F2 / F1

1400 A(67) = S(33) * A(16) * A(27) * F2 * F2

1410 A(68) = S(34) * A(16) * A(28) * F2

1420 A(69) = S(35) * A(16) * A(19) * F2 * F2

1430 A(70) = S(36) * A(16) * A(20) * F2 * F2

1440 A(71) = S(37) * A(16) * A(70)

1450 A(72) = S(38) * A(70) * A(20)

1460 A(73) = S(39) * A(16) * A(21) * F3 * F2 / F1

1470 A(74) = S(40) * A(16) * A(40) * F2 * F2

1480 A(75) = S(41) * A(16) * A(41) * F2

1490 A(76) = S(42) * A(17) * A(27) * F2

1500 A(77) = S(43) * A(17) * A(19) * F2

1510 A(78) = S(44) * A(17) * A(20) * F2

1520 A(79) = S(45) * A(17) * A(21) * F3 * F1 / F2

1530 A(80) = S(9) * A(22) * A(26) * F1

1540 A(23) = S(47) * A(26) * A(12) * F4 / F3

1550 A(81) = S(48) * A(26) * A(23) * F3 / F2

1560 A(82) = S(49) * A(26) * A(81) * F2 / F1

1570 A(83) = S(50) * A(26) * A(82) * F1

1580 A(84) = S(51) * A(15) * A(12) * F4

1590 A(85) = S(52) * A(15) * A(23) * F2 * F3 / F1

1600 A(86) = S(53) * A(15) * A(25) * A(12) * F4 * F2 / F3

1610 A(87) = S(54) * A(15) * A(25) * F2 / F1

1620 A(88) = S(55) * A(16) * A(25) * F2 / F1

1630 A(89) = S(56) * A(16) * A(12) * F4

1640 A(90) = S(57) * A(16) * A(25) * A(12) * F2 * F4 / F3

1650 T(0) = A(13) + A(44) + A(45) + A(46) + A(47) + A(48) + 2 * A(49) + A(50) + A(34) + A(35)

1660 T(1) = A(14) + A(51) + A(52) + A(53) + A(54) + A(55)

1670 T(2) = A(17) + A(76) + A(77) + A(78) + A(79)

1680 T(3) = A(15) + A(56) + A(57) + A(58) + A(59) + A(60) + 2 * A(61) + A(63) + A(64) + A(62) + A(65) + A(36) + A(85) + A(84) + A(86) + A(87)

1690 T(4) = A(16) + A(66) + A(67) + A(68) + A(69) + A(70) + 2 * A(71) + A(73) + A(74) + A(75) + A(72) + A(37) + A(88) + A(89) + A(90)

1700 T(5) = A(18) + A(27) + A(28) + A(29) + A(44) + A(51) + A(56) + A(57) + A(58) + A(66) + A(67) + A(68) + A(76)

1710 T(6) = A(19) + A(38) + A(45) + A(52) + A(59) + A(69) + A(77)

1720 T(7) = A(20) + A(39) + A(60) + A(61) + A(70) + A(71) + A(46) + A(53) + A(78) + 2 * A(62) + 2 * A(72)

1730 T(8) = A(21) + A(40) + A(41) + A(42) + A(47) + A(54) + A(79) + A(63) + A(64) + A(65) + A(73) + A(74) + A(75)

1740 T(9) = A(12) + A(23) + A(81) + A(82) + A(83) + A(84) + A(85) + A(86) + A(89) + A(90) + A(48) + A(49) + A(50) + A(55)

1750 T(10) = A(22) + A(80)

1760 T(11) = A(33) + A(32) + A(30) + A(34) + A(35) + A(36) + A(37)

1762 Rem Cells(11, 26) = "line 1762"

1770 For I1 = 0 To 11

1780 If T(I1) = 0 Then T(I1) = 1E-20

1790 Next I1

1795 Rem rem Cells(11, 27) = "line 1795"

1800 A(13) = A(1) * A(13) / T(0)

1810 A(14) = A(2) * A(14) / T(1)

1820 A(15) = A(3) * A(15) / T(3)

1830 A(16) = A(4) * A(16) / T(4)

1840 A(17) = A(5) * A(17) / T(2)

1850 A(18) = A(6) * A(18) / T(5)

1860 A(19) = A(7) * A(19) / T(6)

1870 A(20) = A(8) * A(20) / T(7)

1880 A(21) = A(9) * A(21) / T(8)

1890 A(22) = A(10) * A(22) / T(10)

1900 A(12) = A(11) * A(12) / T(9)

1910 A(33) = A(31) * A(33) / T(11)

1912 Rem Cells(11, 28) = "line 1912"

1920 S1 = (A(25) + A(26)) / F1 + A(13) + A(14) + A(17) + A(22) + A(91) + A(44) + A(45) + A(46) + A(34) + A(51) + A(52) + A(53) + A(76) + A(77)

1930 S1 = S1 + A(78) + A(56) + A(58) + A(63) + A(65) + A(85) + A(87) + A(28) + A(32) + A(38) + A(39) + A(41) + A(82)

1940 S2 = 4 * (A(15) + A(16) + A(19) + A(20) + A(33) + A(47) + A(49) + A(54) + A(79) + A(84) + A(50) + A(89) + A(27) + A(40) + A(81) + A(61) + A(71) + A(62) + A(72))

1950 S3 = 9 * (A(18) + A(21) + A(48) + A(55) + A(23) + A(86) + A(90))

1960 S4 = 16 * A(12)

1970 S5 = (S1 + S2 + S3 + S4) / 2

1980 If S5 > 1 Then S5 = 1

1990 If S5 < 0.000001 Then S5 = 0.000001 Else GoTo 2000

2000 S6 = Sqr(S5)

2010 F1 = Exp(0 - 1.20218 * ((S6 / (1 + S6)) - 0.285 * S5))

2020 F2 = F1 ^ 4

2030 F3 = F1 ^ 9

2040 F4 = F1 ^ 16

2050 If A(15) = 0 Then GoTo 2070

2060 If Abs((A(15) - O0) / A(15)) > 0.0001 Then GoTo 2160

2070 If A(16) = 0 Then GoTo 2090

2080 If Abs((A(16) - O1) / A(16)) > 0.0001 Then GoTo 2160

2090 If A(18) = 0 Then GoTo 2110

2100 If Abs((A(18) - O2) / A(18)) > 0.0001 Then GoTo 2160

2110 If A(20) = 0 Then GoTo 2130

2120 If Abs((A(20) - O3) / A(20)) > 0.0001 Then GoTo 2160

2130 If A(21) = 0 Then GoTo 2150

2140 If Abs((A(21) - O4) / A(21)) > 0.0001 Then GoTo 2160

2150 GoTo 2231

2160 O0 = A(15)

2170 O1 = A(16)

2180 O2 = A(18)

2190 O3 = A(20)

2200 O4 = A(21)

2210 Next i

2231 A(93) = S5

2232 A(94) = F1

2234 A(95) = F2

2236 A(96) = F3

2238 A(97) = F4

2240 Rem Cells(11, 29) = "line 2240"

2250 Rem GoTo 3000

2258 Rem print , P$, DAT$: rem print

2260 Rem print , "NA", A(1), "[NAHPP]", A(50)

2270 Rem print , "K", A(2), "[KHPO4]", A(51)

2280 Rem print , "CA", A(3), "[KSO4]", A(52)

2290 Rem print , "MG", A(4), "[KOX]", A(53)

2300 Rem print , "NH4", A(5), "[KCIT]", A(54)

2310 Rem print , "PO4", A(6), "[KPP]", A(55)

2320 Rem print , "SO4", A(7), "[CAPO4]", A(56)

2330 Rem print , "OX", A(8), "[CAHPO4]", A(57)

2340 Rem print , "CIT", A(9), "[CAH2P04]", A(58)

2350 Rem print , "U", A(10), "[CASO4]", A(59)

2360 Rem print , "PP", A(11), "[CAOX]", A(60)

2370 Rem print , "[PP]", A(12), "[CA2OX]", A(61)

2380 Rem print , "[NA]", A(13), "[CAOX2]", A(62)

2390 Rem print , "[K]", A(14), "[CACIT]", A(63)

2400 Rem print , "[CA]", A(15), "[CAHCIT]", A(64)

2410 Rem print , "[MG]", A(16), "[CAH2CIT]", A(65)

2420 Rem print , "[NH4]", A(17), "[MGPO4]", A(66)

2430 Rem print , "[PO4]", A(18), "[MGHPO4]", A(67)

2440 Rem print , "[SO4]", A(19), "[MGH2PO4]", A(68)

2450 Rem print , "[OX]", A(20), "[MGSO4]", A(69)

2460 Rem print , "[CIT]", A(21), "[MGOX]", A(70)

2470 Rem print , "[HU]", A(22), "[MG2OX]", A(71)

2480 Rem print , "[HPP]", A(23), "[MGOX2]", A(72)

2490 Rem print , "PH", A(24), "[MGCIT]", A(73)

2500 Rem print , "(OH)", A(25), "[MGHCIT]", A(74)

2510 Rem print , "(H)", A(26), "[MGH2CIT]", A(75)

2520 Rem print , "[HPO4]", A(27), "[NH4HPO4]", A(76)

2530 Rem print , "[H2PO4]", A(28), "[NH4SO4]", A(77)

2540 Rem print , "[H3PO4]", A(29), "[NH4OX]", A(78)

2550 Rem print , "[H2CO3]", A(30), "[NH4CIT]", A(79)

2560 Rem print , "CO2", A(31), "[H2U]", A(80)

2570 Rem print , "[HCO3]", A(32), "[H2PP]", A(81)

2580 Rem print , "[CO3]", A(33), "[H3PP]", A(82)

2590 Rem print , "[NACO3]", A(34), "[H4PP]", A(83)

2600 Rem print , "[NA2CO3]", A(35), "[CAPP]", A(84)

2610 Rem print , "[CACO3]", A(36), "[CAHPP]", A(85)

2620 Rem print , "[MGCO3]", A(37), "[CAOHPP]", A(86)

2630 Rem print , "[HSO4]", A(38), "[CAOH]", A(87)

2640 Rem print , "[HOX]", A(39), "[MGOH]", A(88)

2650 Rem print , "[HCIT]", A(40), "[MGPP]", A(89)

2660 Rem print , "[H2CIT]", A(41), "[MGOHPP]", A(90)

2670 Rem print , "[H3CIT]", A(42), "[CL]", A(91)

2680 Rem print , "[H2U]", A(43), "CYCLES", A(92)

2690 Rem print , "[NAHPO4]", A(44), "I.S.", A(93)

2700 Rem print , "[NASO4]", A(45), "F1", A(94)

2710 Rem print , "[NAOX]", A(46), "F2", A(95)

2720 Rem print , "[NACIT]", A(47), "F3", A(96)

2730 Rem print , "[NAPP]", A(48), "F4", A(97)

2740 Rem print , "[NA2PP]", A(49)

2750 Rem print , "[NAHPP]", A(50)

2760 Rem rem print

2770 Rem print , , "SS", "DG"

2780 A(100) = A(60) / 0.00000616

2790 A(101) = A(15) * A(27) * F2 * F2 / 0.000000237

2800 X1 = F2 * A(15) * 1000

2810 X2 = F3 * A(18) * 10000000000#

2820 A(102) = (X1 ^ 5) * (X2 ^ 3) * A(25) / 1.45E-14

2830 A(103) = F1 * F2 * F3 * A(16) * A(17) * A(18) / 0.000000000000115

2840 A(104) = A(80) / 0.000261

2850 A(105) = F1 * F1 * A(22) * A(13) / 0.0000279

2860 A(106) = F1 * F1 * A(22) * A(17) / 0.000036

2870 A(107) = F1 * F1 * A(22) * A(14) / 0.0000963

2880 Rem A(108) = 1.2935 * Log(A(100))

2890 Rem A(109) = 1.2935 * Log(A(101))

2900 Rem A(110) = 0.28744 * Log(A(102))

2910 Rem A(111) = 0.8623 * Log(A(103))

2920 Rem A(112) = 2.587 * Log(A(104))

2930 Rem A(113) = 1.2935 * Log(A(105))

2940 Rem A(114) = 1.2935 * Log(A(106))

2950 Rem A(115) = 1.2935 * Log(A(107))

3004 Cells(j - 20, 5) = (A(100))

3005 Rem Cells(10, 15) = "caox"

3006 Cells(j - 20, 8) = (A(103))

3008 Rem Cells(10, 16) = "bru"

3010 Rem print , "BRU", A(101), A(109)

3020 Rem print , "HAP", A(102), A(110)

3030 Rem print , "STRU", A(103), A(111)

3040 Rem print , "HU", A(104), A(112)

3050 Rem print , "NAU", A(105), A(113)

3060 Rem print , "NH4U", A(106), A(114)

3070 Rem print , "KU", A(107), A(115)

3072 j = j + 1

3073 If j > 40 Then GoTo 3250

3074 i = j

3075 Rem Cells(8, 17) = i: Cells(8, 18) = j

3076 GoTo 760

3080 Rem rem print  : rem print  : rem print  : rem print

3100 Rem CLOSE

3240 Rem CLOSE

3250 End

End Sub
